# Long-term adjuvant metronomic chemotherapy in a dog with recurrent maxillofacial osteosarcoma

**DOI:** 10.17221/43/2022-VETMED

**Published:** 2023-05-25

**Authors:** Min-Jung Jung, Keun-Young Yoon, Yun-Mi Kim, Jong-Sun Lee, Joo-Won Choi, Ji-Hyun Kim, Hun-Young Yoon, Jung-Hyun Kim

**Affiliations:** ^1^Department of Veterinary Internal Medicine, College of Veterinary Medicine, Konkuk University, Gwangjin-gu, Seoul, Republic of Korea; ^2^Department of Veterinary Surgery, College of Veterinary Medicine, Konkuk University, Gwangjin-gu, Seoul, Republic of Korea; ^3^KU Center for Animal Blood Medical Science, Gwangjin-gu, Seoul, Republic of Korea

**Keywords:** adverse effects, cyclophosphamide, melanoma, piroxicam, survival time

## Abstract

Osteosarcoma (OSA) is the most common malignant bone tumour in dogs; however, OSA of the maxilla is uncommon compared to appendicular OSA. Oral melanoma also commonly occurs in dogs with frequent distant metastasis. The role of adjuvant chemotherapy has been questioned in maxillary OSA and melanoma. A 17-year-old English Cocker Spaniel was referred with a growing mass on the right maxilla and a right lower lip mass. Osteosarcoma was diagnosed after partial maxillectomy, and the right lower lip mass was diagnosed as oral melanoma. Metronomic chemotherapy (MC) was performed, and the number of doses was tapered due to side effects at 5 weeks after initiation of MC. After 130 weeks of MC, chemotherapy was suspended due to kidney disease. After the suspension of chemotherapy, findings suggesting recurrence and metastasis were detected. The dog suddenly died 193 weeks after surgery, which was 8–14 times longer than the expected survival time. To the best of our knowledge, this is the first case report of successful long-term combination therapy, including surgery and MC, in a dog with maxillary OSA and lip melanoma. Our results show that the survival time can be greatly extended if MC is performed with proper management.

Osteosarcoma (OSA) is the most common malignant bone tumour in dogs, most frequently affecting the appendicular skeleton (Szewczyk et al. 2015; Matsuyama et al. 2018). Osteosarcoma of the maxilla, mandible, or calvarium is comparatively uncommon, representing up to 12.4% of reported osteosarcoma cases (Farcas et al. 2014; Selmic et al. 2014). The standard treatment for osteosarcoma is complete surgical resection; adjuvant treatments, including radiation therapy and chemotherapy, can be attempted if incomplete resection is achieved (Farcas et al. 2014).

Oral melanoma is the most fatal form of canine melanoma, and surgical excision is considered a standard treatment. Radiotherapy and chemotherapy have been used as adjuvant therapy, and immunotherapy, such as melanoma vaccine, can also be attempted for advanced stage cases. However, the efficacy of treatment is poor (Silveira et al. 2020; Stevenson et al. 2023). The location, initial tumour size, and especially clinical stage are important prognostic indicators (Silveira et al. 2020).

Metronomic chemotherapy (MC) involves long-term administration of chemotherapeutic agents at low doses without drug-free breaks (Romiti et al. 2013; Matsuyama et al. 2018). It inhibits both local and systemic vasculogenic effects of the tumour by inducing apoptosis of vascular endothelial cells and modulating angiogenic factors (London et al. 2015; Gaspar et al. 2018). It also depletes immunosuppressive regulatory T cells, which control anticancer immune reactions (Romiti et al. 2013; Gaspar et al. 2018). These combined antineoplastic effects make MC more effective in osteosarcoma and melanoma, which have high angiogenic capacity (Matsuyama et al. 2018).

Various adjuvant chemotherapies, including chemotherapy with cisplatin, carboplatin, and cyclophosphamide, as well as combinations of these, for patients with incompletely resected appendicular osteosarcoma have been reported to extend the mean survival time from 3–5 months to 8–12 months (Matsuyama et al. 2018; Poon et al. 2020).

However, the role of adjuvant chemotherapy has been questioned in maxillary osteosarcoma (Selmic et al. 2014). To our knowledge, this is the longest surviving case of osteosarcoma case involving combination therapy, including surgery and MC, demonstrating a survival of approximately 44 months, which is 8–14 times longer than that of surgically treated OSA cases (Matsuyama et al. 2018; Poon et al. 2020).

## Case description

**Figure 1:**
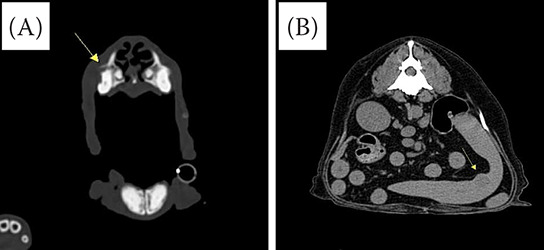
Computed tomography of the (A) maxilla and (B) abdomen (A) Periapical alveolar bone shows mild lytic appearance [Rt. (arrow) > Lt.]. There is no evidence of patency with the adjacent nasal cavity. (B) Round hypo-iso attenuated nodule in the splenic body (nodule size: 10.31 × 11.02 × 9.9 mm; arrow)

A 17-year-old spayed female English Cocker Spaniel was admitted to our hospital with a growing mass on the right maxilla. The mass had recurred due to incomplete surgical removal. The tumour showed aggressive enlargement over 4 months. On computed tomography, periosteal reaction was detected in the bilateral maxillary canine teeth and periapical alveolar bone showed mild lytic appearance. Hypo-iso attenuated nodules were detected in the splenic body and several attenuated foci were detected in the lung (Figure 1).

**Figure 2:**
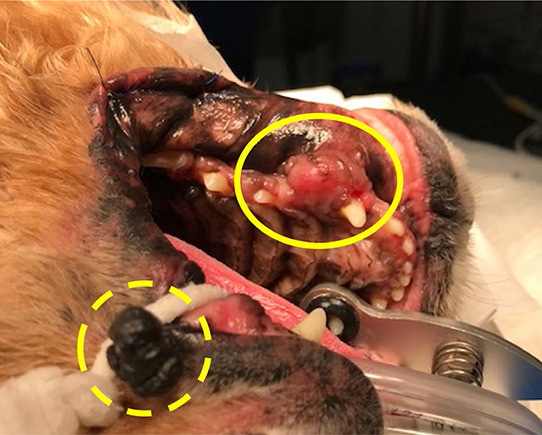
Patient at the time of the surgery Note the right maxillary gingival mass (size: 2.5 cm × 2.5 cm × 2.3 cm) (yellow circle) and right lower lip mass (size: 1.5 cm × 1 cm; yellow dotted circle)

No specific findings were observed in the lymph nodes. Right-sided partial maxillectomy (third incisor to first premolar) was performed to remove a right maxillary gingival mass (2.5 × 2.5 × 2.3 cm) and a right lower lip mass (1.5 × 1 cm) (Figure 2).

**Figure 3:**
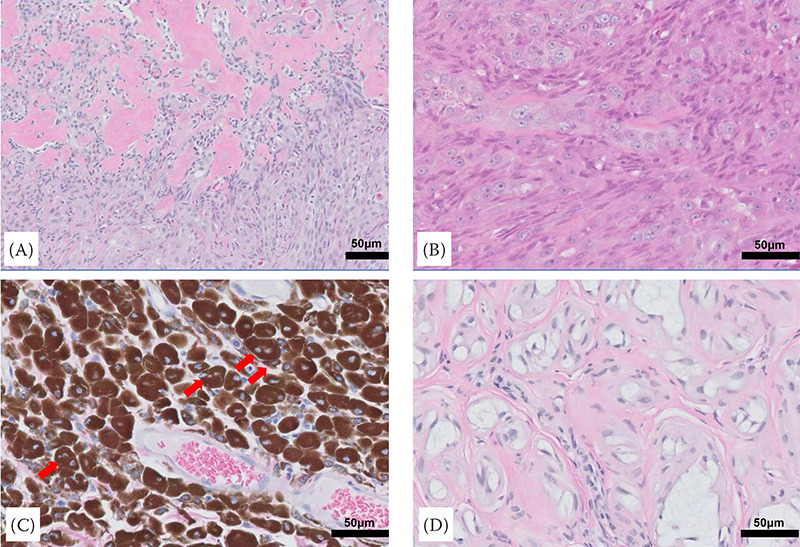
Histopathology of osteosarcoma resected from the right gingiva (A, B) and oral melanoma resected from the right lower lip (C, D). The osteosarcoma is completely excised with a narrow margin (A) Right gingival mass. The mass is poorly demarcated, unencapsulated, and infiltrative. (B) Right gingival mass. Marked anisocytosis and anisokaryosis are seen. The mitotic index is 3 per 10 high power fields. (C) Right lower lip mass. Neoplastic cells have rare dark brown to black intracytoplasmic pigment (melanin). Red arrows indicate neoplastic cells with 2–3 distinct nucleoli. Vascular invasion is not observed. (D) Right lower lip mass (melanin bleach). Neoplastic melanocytes have mild cellular atypical features, mild anisocytosis, and anisokaryosis *Haematoxylin and eosin stain; scale bar = 50 μm

Based on histopathologic findings (IDEXX Laboratories, Seoul, Republic of Korea), the right gingival mass was confirmed to be an osteosarcoma stage IIB (G2 T2 M0) without metastasis and the right lower lip mass was confirmed to be a melanoma stage I (T1 N0 M0) with a complete margin (Figure 3).

Adjuvant chemotherapy was planned because OSA showed a narrow margin, there was a history of recurrence, and there was a concurrent diagnosis of melanoma of the ipsilateral lip.

At the time of presentation for first chemotherapy, blood examination revealed no abnormal findings, with all values being within the reference range. Radiographic examination showed no nodules in the chest and abdomen. Ultrasonography showed a round shaped hypoechoic nodule in the splenic head region (10.31 × 11.02 × 9.9 mm). Because there was no blood flow on the Colour Doppler, it was considered a nodular hyperplasia.

Therefore, MC with piroxicam [Piroxicam 0.3 mg/kg per os (p.o.) once daily], cyclophosphamide (Alkyloxan 10 mg/m^2^ p.o. once daily), and misoprostol (5 μg/kg p.o. once daily) was initiated. Following chemotherapy, side effects and metastasis were evaluated every 2 weeks, followed by every month and 3 months, as there were no significant changes.

At the 5^th^ week after the initial MC, the urinary bladder wall was irregular and thickened (3 mm), more than twice the size at initial presentation (1.3 mm). Although there were no related clinical symptoms, cyclophosphamide dose was changed from once a day to once every 2 days because wall thickening could be an initial symptom of cystitis, which is a side effect of cyclophosphamide. Three weeks after the cyclophosphamide dose tapering, bladder ultrasonography showed resolution and administration was maintained at every 2 days.

Follow-up abdominal ultrasound examination was performed every 3 months for the assessment of metastasis status and any change in the size of nodules in the spleen. The size of the splenic head hypoechoic nodule (7.2 × 7.5 mm) remained unchanged for 29 months. At week 125, a new nodule (2.8 × 4.3 mm) was detected in the splenic tail, which was hyperechoic, heterogenous, and negative on E-flow.

**Figure 4:**
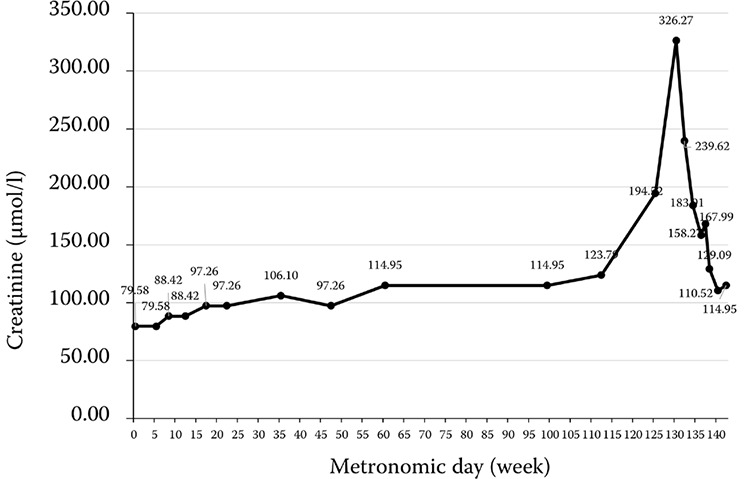
Serum creatinine levels (μmol/l) during the 143-week course of metronomic chemotherapy Creatinine levels increased gradually following the start of metronomic chemotherapy, and suddenly increased to 326.27 μmol/l in the 130^th^ week. A diagnosis of chronic kidney disease IRIS stage III of IV was made, which corresponded to VCOG grade III and metronomic chemotherapy was suspended. The arrow on the X-axis indicates the period during which metronomic chemotherapy was applied

At week 112, serum chemistry showed elevated urea nitrogen [2.28 mmol/l (RI, 0.39–1.50 mmol/l)] and creatinine [123.79 μmol/l (RI, 44.2–159.16)], levels, and chronic kidney disease (IRIS stage II of IV) with borderline proteinuria [urine protein-to-creatinine ratio (UPC), 0.35; RI, 0.2–0.5] was diagnosed. However, as there were no clinical symptoms associated with renal failure, an appropriate drinking amount was recommended, and renal function was monitored monthly. After 18 weeks, biochemistry revealed sudden elevated creatinine levels (326.27 μmol/l), which were more than twice the upper limit of normal. Therefore, MC was suspended because the elevated creatinine levels corresponded to grade III adverse effects according to the VCOG criteria (LeBlanc et al. 2021). Further chemotherapy was not possible due to renal failure, and MC was suspended (Figure 4).

At the 143^rd^ week of MC, 3 months after MC suspension, the dog was admitted for check-up. Physical examination showed gingival hyperplasia at the front maxilla and left maxilla. Complete blood count revealed moderate anaemia [haematocrit, 27.6% (RI, 37.3–61.7%)], and biochemistry revealed hyperphosphatemia [7.5 mmol/l (RI, 3.5–5.8 mmol/l)] and elevated d-dimer levels [423.51 ng/ml (RI, 50–250 ng/ml)]. Radiography showed a new nodule (4.6 × 4.6 mm) in the left cranial lung lobe. Ultrasonography revealed a new hypoechoic nodule (5.6 × 7.6 mm) in the splenic tail, which was positive on E-flow, unlike the previous splenic nodules. A biopsy for gingival hyperplasia and a metastasis evaluation could not be performed because of high risk of anaesthesia and poor patient compliance. Anti-cancer drugs were not recommended as the patient was diagnosed with end-stage chronic kidney disease. The dog showed a gradual deterioration in energy and loss of appetite, and he was finally referred to a local animal hospital for hospice care.

Ten months thereafter, the dog was to visit the hospital due to persistent nasal discharge, anorexia, and lethargy. However, he suddenly died the day before the visit, which was 44 months postoperatively, 43 months after MC initiation, and 13 months after MC suspension. Post-mortem examination was declined.

## DISCUSSION

Osteosarcoma is highly aggressive and micrometastasis occurs in 90% of dogs (Szewczyk et al. 2015). Comparatively, dogs with osteosarcoma of the maxilla and mandible commonly die due to the local invasion as opposed to metastatic disease, which is the usual cause of death in appendicular osteosarcoma cases (Selmic et al. 2014; Coyle et al. 2015). Moreover, complete surgical resection of maxillary OSA is more difficult than amputation of the appendicular skeleton, leading to a higher local recurrence rate of 58% (Mouser et al. 2006; Selmic et al. 2014). Local tumour recurrence and subsequent regional disease failure might lead to early death, and the true metastatic potential of OSA arising from maxilla is underestimated (Selmic et al. 2014; Coyle et al. 2015). In this case, partial maxillectomy was performed due to the local recurrence of maxillary OSA, and the right lower lip mucosal melanoma was completely removed. Although oral melanoma is expected to have a relatively better prognosis due to its small size, oral malignant melanoma is associated with frequent distant metastasis (Silveira et al. 2020). It is also important to secure a wide resection in OSA due to its high local recurrence rate (Farcas et al. 2014; Selmic et al. 2014); however, a narrow margin was secured and the dog already had a history of recurrence. Therefore, adjuvant chemotherapy was needed for management of recurrence and metastasis of maxillary OSA and lip melanoma.

In veterinary medicine, MC typically comprises low-dose cyclophosphamide and piroxicam (London et al. 2015; Gaspar et al. 2018). Cyclophos-phamide is a nitrogen mustard alkylating agent, which is a widely used chemotherapeutic agent in MC (Penel et al. 2012). It successfully manages multiple malignancies, with strong immunomodulatory and anti-angiogenic abilities (Gaspar et al. 2018). Piroxicam is also commonly used in veterinary metronomic protocols. COX-2 expression in circulating endothelial precursors (CEPs) is important for tumour cell survival. Therefore, inhibiting COX-2 may reduce the ability of CEPs to proliferate and survive in the tumour microenvironment (London et al. 2015).

The most concerning toxicity associated with cyclophosphamide is sterile haemorrhagic cystitis, which is caused by cyclophosphamide metabolites and acrolein that irritate the bladder endothelium (Harper and Blackwood 2017; Matsuyama et al. 2018). It has a prevalence rate of 5–25% of dogs treated with cyclophosphamide, usually after an average of 18 weeks of therapy (Chan et al. 2016). In this case, bladder wall thickness and irregularity at the 5^th^ week were considered early symptoms of sterile haemorrhagic cystitis, which were resolved by reducing the chemotherapeutic dose. This case highlighted the importance of regular urinalysis and urinary bladder ultrasonography for the early detection of sterile haemorrhagic cystitis to prevent the development of subsequent serious diseases and suspension of MC.

Gastrointestinal (GI) side effects and renal toxicosis may also occur due to long-term use of cyclophosphamide and piroxicam (Eichstadt et al. 2017). In our case, GI side effects were not observed; however, creatinine levels increased gradually following the start of MC. Subsequently, the creatinine levels suddenly increased, and the dog was diagnosed with CKD IRIS stage III of IV with proteinuria (UPC, 0.89) and chemotherapy was ceased. Thereafter, renal failure was worsening, hyperphosphataemia and anaemia were detected, and reuptake of chemotherapeutic agents was rejected.

Metastatic evaluation is also important during MC. During chemotherapy, neither recurrence nor distant metastasis findings were detected. However, at 3 months after chemotherapy suspension, hyperplasia of the maxillary gingiva and novel nodules in the lung and spleen were detected. The previously detected nodules showed no blood flow on Colour Doppler. However, these new nodules were positive on E-flow and were considered metastasis of the OSA or melanoma.

After 10 months of chemotherapy suspension, the chief complaint was yellow nasal discharge without coughing and lethargy. Nasal metastatic OSA was thought to be the most likely cause considering other metastasis suspicious nodules. This supports the previous argument that metastasis in maxillary OSA may be underestimated due to early death. This case leads us to the conclusion that maxillary OSA produces metastasis as well as local recurrence, and MC is effective in preventing metastasis and recurrence of OSA. Unfortunately, the exact metastasis evaluation using CT or biopsy could not be confirmed because of the anaesthesia risk associated with severe kidney disease and the dog’s low compliance.

To the best of our knowledge, this is the first case report of successful long-term medical management of maxillary OSA and ipsilateral lip melanoma in a dog, demonstrating that the survival time can be greatly extended if MC is performed with proper management. We suggest that for maxillary OSA and lip melanoma patients, it is important to apply MC after surgery as adjuvant chemotherapy for preventing metastasis and recurrence. It is also important to manage MC with proper adverse effect evaluation for long-term survival.

## References

[R1] Chan CM, Frimberger AE, Moore AS. Incidence of sterile hemorrhagic cystitis in tumor-bearing dogs concurrently treated with oral metronomic cyclophosphamide chemotherapy and furosemide: 55 cases (2009–2015). J Am Vet Med Assoc. 2016 Dec 15;249(12):1408-14. 27901449 10.2460/javma.249.12.1408

[R2] Coyle VJ, Rassnick KM, Borst LB, Rodriguez CO Jr, Northrup NC, Fan TM, Garrett LD. Biological behaviour of canine mandibular osteosarcoma. A retrospective study of 50 cases (1999–2007). Vet Comp Oncol. 2015 Jun;13(2):89-97. 10.1111/vco.12020 23410097

[R3] Eichstadt LR, Moore GE, Childress MO. Risk factors for treatment-related adverse events in cancer-bearing dogs receiving piroxicam. Vet Comp Oncol. 2017 Dec;15(4):1346-53. 27714960 10.1111/vco.12276

[R4] Farcas N, Arzi B, Verstraete FJ. Oral and maxillofacial osteosarcoma in dogs: A review. Vet Comp Oncol. 2014 Sep;12(3):169-80. 22935032 10.1111/j.1476-5829.2012.00352.x

[R5] Gaspar TB, Henriques J, Marconato L, Queiroga FL. The use of low-dose metronomic chemotherapy in dogs-insight into a modern cancer field. Vet Comp Oncol. 2018 Mar;16(1):2-11. 28317239 10.1111/vco.12309

[R6] Harper A, Blackwood L. Toxicity of metronomic cyclophosphamide chemotherapy in a UK population of cancer-bearing dogs: A retrospective study. J Small Anim Pract. 2017 Apr;58(4):227-30. 28133740 10.1111/jsap.12635

[R7] LeBlanc AK, Atherton M, Bentley RT, Boudreau CE, Burton JH, Curran KM, Dow S, Giuffrida MA, Kellihan HB, Mason NJ, Oblak M, Selmic LE, Selting KA, Singh A, Tjostheim S, Vail DM, Weishaar KM, Berger EP, Rossmeisl JH, Mazcko C. Veterinary Cooperative Oncology Group-Common Terminology Criteria for Adverse Events (VCOG-CTCAE v2) following investigational therapy in dogs and cats. Vet Comp Oncol. 2021 Jun;19(2):311-52. 33427378 10.1111/vco.12677 PMC8248125

[R8] London CA, Gardner HL, Mathie T, Stingle N, Portela R, Pennell ML, Clifford CA, Rosenberg MP, Vail DM, Williams LE, Cronin KL, Wilson-Robles H, Borgatti A, Henry CJ, Bailey DB, Locke J, Northrup NC, Crawford-Jakubiak M, Gill VL, Klein MK, Ruslander DM, Thamm DH, Phillips B, Post G. Impact of toceranib/piroxicam/cyclophosphamide maintenance therapy on outcome of dogs with appendicular osteosarcoma following amputation and carboplatin chemotherapy: A multi-institutional study. PLoS One. 2015 Apr 29;10(4):e0124889. 25923466 10.1371/journal.pone.0124889 PMC4414350

[R9] Matsuyama A, Schott CR, Wood GA, Richardson D, Woods JP, Mutsaers AJ. Evaluation of metronomic cyclophosphamide chemotherapy as maintenance treatment for dogs with appendicular osteosarcoma following limb amputation and carboplatin chemotherapy. J Am Vet Med Assoc. 2018 Jun 1;252(11):1377-83. 29772973 10.2460/javma.252.11.1377

[R10] Mouser P, Cole A, Lin TL. Maxillary osteosarcoma in a prairie dog (Cynomys ludovicianus). J Vet Diagn Invest. 2006 May;18(3):310-2. 16789726 10.1177/104063870601800317

[R11] Penel N, Adenis A, Bocci G. Cyclophosphamide-based metronomic chemotherapy: After 10 years of experience, where do we stand and where are we going? Crit Rev Oncol Hematol. 2012 Apr;82(1):40-50. 21641231 10.1016/j.critrevonc.2011.04.009

[R12] Poon AC, Matsuyama A, Mutsaers AJ. Recent and current clinical trials in canine appendicular osteosarcoma. Can Vet J. 2020 Mar;61(3):301-8. 32165755 PMC7020630

[R13] Romiti A, Cox MC, Sarcina I, Di Rocco R, D’Antonio C, Barucca V, Marchetti P. Metronomic chemotherapy for cancer treatment: A decade of clinical studies. Cancer Chemother Pharmacol. 2013 Jul;72(1):13-33. 23475105 10.1007/s00280-013-2125-x

[R14] Selmic LE, Lafferty MH, Kamstock DA, Garner A, Ehrhart NP, Worley DR, Withrow SJ, Lana SE. Outcome and prognostic factors for osteosarcoma of the maxilla, mandible, or calvarium in dogs: 183 cases (1986–2012). J Am Vet Med Assoc. 2014 Oct 15;245(8):930-8. 25285935 10.2460/javma.245.8.930

[R15] Silveira TL, Veloso ES, Goncalves INN, Costa RF, Rodrigues MA, Cassali GD, Del Puerto HL, Pang LY, Argyle DJ, Ferreira E. Cyclooxygenase-2 expression is associated with infiltration of inflammatory cells in oral and skin canine melanomas. Vet Comp Oncol. 2020 Dec;18(4):727-38. 32323423 10.1111/vco.12601

[R16] Stevenson VB, Klahn S, LeRoith T, Huckle WR. Canine melanoma: A review of diagnostics and comparative mechanisms of disease and immunotolerance in the era of the immunotherapies. Front Vet Sci. 2023 Jan 6;9:1046636. 36686160 10.3389/fvets.2022.1046636 PMC9853198

[R17] Szewczyk M, Lechowski R, Zabielska K. What do we know about canine osteosarcoma treatment? Review. Vet Res Commun. 2015 Mar;39(1):61-7. 25422073 10.1007/s11259-014-9623-0 PMC4330401

